# Quantification of 3D Kinematic Measurements for Knee Flexion and Tibial Rotation Using an IMU-Based Sensor and Ultrasound Imaging System: A Cadaveric Study

**DOI:** 10.3390/s25134211

**Published:** 2025-07-06

**Authors:** Hamid Rahmatullah Bin Abd Razak, Nicolas Chua, Kah Weng Lai

**Affiliations:** 1 Department of Orthopaedic Surgery, Sengkang General Hospital, Singapore 544886, Singapore; hamidrazak@gmail.com; 2 SingHealth Duke-NUS Musculoskeletal Sciences Academic Clinical Programme, Academia Level 4, 20 College Road, Singapore 169856, Singapore; 3 Total Orthopaedic Care & Surgery, Novena Medical Centre, 10 Sinaran Drive, #09-24, Singapore 307506, Singapore; 4 College of Design and Engineering, National University of Singapore, 9 Engineering Drive 1, #07-26 EA, Singapore 117575, Singapore; nicolaschua@u.nus.edu; 5 Island Orthopaedics, Mount Elizabeth Novena Specialist Centre, 38 Irrawaddy Rd, #05-42, Singapore 329563, Singapore; 6 PreciX Pte Ltd., 2 College Rd, Singapore 169850, Singapore

**Keywords:** knee kinematics, IMU, ultrasound, wearable sensors, motion tracking, cadaveric study, rotational stability, anterior cruciate ligament, ACL, remote rehabilitation

## Abstract

Knee rotational stability is crucial for anterior cruciate ligament (ACL) procedures, yet, current clinical assessments are subjective and lack precision. This study evaluates the accuracy and repeatability of the GATOR system, developed by PreciX Pte Ltd. and integrating ultrasound with inertial measurement units (IMUs), against a reference IMU (Xsens DOTS) for measuring knee flexion and rotation in six cadaveric specimens secured in an Oxford Knee Jig. Two experiments were conducted: (A) knee flexion from 0° to 120°, and (B) internal/external rotation at 0°, 30°, 60°, 90°, and 120° flexion. Analysis using Bland–Altman plots, root mean square error (RMSE: 3.93° for internal rotation, 6.90° for external rotation), mean biases, and paired *t*-tests (Bonferroni corrected) revealed that GATOR recorded lower peak flexion angles (91.49–114.65°) compared to the reference (110.31–118.49°). For rotation, internal rotation showed narrower limits of agreement than external rotation (biases: 1.91–6.88°). Over 60% of trials had errors < 5°, and 80% < 10°, indicating good agreement. Despite no isolated comparison of GATOR’s ultrasound component, findings suggest reduced soft tissue artifact due to bone-referenced sensor alignment. With optimal placement (10–15 cm from the knee center), GATOR shows promise in ACL assessment and remote rehabilitation.

## 1. Introduction

### 1.1. Clinical Significance of Knee Rotational Stability

Knee rotational stability is a key biomechanical parameter in both pre-operative planning and post-operative evaluation of anterior cruciate ligament (ACL) injuries. The ACL plays a pivotal role in resisting internal tibial rotation and anterior tibial translation, contributing to overall joint stability [1,2,3,4]. When this stability is compromised, persistent rotational laxity following ACL reconstruction correlates directly with poor functional outcomes and increased risk of graft failure and post-traumatic osteoarthritis (PTOA) [3,4]. The consequences extend beyond the primary injury, as rotational instability precipitates secondary injuries including meniscal tears and cartilage degeneration, which can accelerate joint degeneration and compromise long-term function [5]. As such, precise measurement of tibial rotation is critical for guiding surgical decision-making, evaluating graft tensioning, and monitoring rehabilitation.

### 1.2. Limitations of Current Assessment Methods

Despite its clinical importance, current assessment methods for rotational stability are primarily qualitative. Commonly used tools such as the pivot shift and Lachman tests rely heavily on clinician skill, are prone to high inter- and intra-rater variability, and lack precise quantification [6,7,8,9,10,11,12,13,14,15]. This variability has been systematically documented by Lange et al. who revealed substantial inconsistencies in physical examination tests for ACL rupture diagnosis. Their analysis showed the Lachman test demonstrates wide inter-rater reliability variation (Cohen’s kappa: 0.19–0.82, spanning slight to substantial agreement), while Intraclass Correlation Coefficients reach 0.92 for ordinal grading. The pivot shift test exhibited comparable variability in reliability (proportion of agreement: 0.32–0.82) [15].

These inconsistencies arise from multiple examiner-dependent factors, including charting methodology, experience level, and specialization degree, all of which significantly compromise inter-rater reliability for the pivot shift test [16]. Geographic variations further compound these issues; a global survey documented distinct technique differences among orthopedic surgeons across Asia, North America, and Europe’s northern, southern, and eastern regions [17]. These challenges in standardizing the assessment to reduce variability translate directly into clinical practice. Patients often receive evaluations from multiple clinicians, each with different training and experience levels, during referrals or follow-up visits. When these clinicians reach different diagnostic conclusions, treatment planning potentially becomes compromised and erodes patient confidence.

These limitations have spurred ongoing interest in quantitative, objective methods to assess knee joint motion in vivo.

### 1.3. Challenges with Existing Measurement Technologies

Gold-standard stereophotogrammetric 3D measurement methods face several drawbacks in clinical translation. Bone pin-based methods, while highly accurate, are invasive and not viable for routine assessments [18]. Optical motion capture systems are noninvasive but susceptible to soft tissue artifact (STA), as skin-mounted markers do not reflect true bone movement during dynamic tasks [12,13,14,15,16,17,18,19,20,21,22,23,24]. Biplanar radiography mitigates soft tissue artifacts (STA) [25] but requires ionizing radiation, specialized infrastructure, and trained personnel, limiting its accessibility and repeatability [26,27,28,29]. Imaging modalities such as MRI and CT offer excellent anatomical detail but are non-portable, not suitable for dynamic tracking, and costly, with CT also introducing radiation exposure [26,27,28,29].

Wearable inertial measurement units (IMUs) have emerged as a potential alternative [30,31]. They offer portability and are not restricted by strict measurement fields [32], and sensor-to-segment calibration can reduce inter-operator variability compared to purely marker-based systems [33,34]. However, IMUs can still be affected by soft tissue artifacts (STA), as they move in tandem with soft tissue rather than bone [35,36,37,38,39].

Seeking to overcome these limitations, the GATOR system integrates ultrasound imaging with IMUs in a novel sensor-fusion approach. It objectively measures knee rotational stability by capturing 3D kinematic measurements of the knee joint. Through this dual-modality technique, GATOR minimizes STA and avoids many of the drawbacks inherent in many state-of-the-art optical or radiographic methods, promising greater accuracy in quantifying the 3D kinematics of knee rotation.

### 1.4. Objective

This cadaveric study investigates the accuracy, and repeatability of GATOR’s knee kinematic measurements for clinical application, benchmarking GATOR outputs against a reference IMU system. It aligns with the growing need for objective, portable tools that can be deployed in surgical, clinical, and remote rehabilitation environments.

## 2. Materials and Methods

### 2.1. GATOR System Description

The GATOR system, developed by PreciX Pte Ltd. (Singapore), is a dual-modality wearable sensor platform that integrates ultrasound imaging with inertial measurement units (IMUs). It is specifically designed to overcome the limitations of conventional IMU-only systems by reducing soft tissue artifact (STA), which commonly distorts kinematic measurements due to sensor displacement relative to bone. The GATOR system comprises two units: a proximal thigh unit that houses both an IMU and an ultrasound module, and a distal tibial unit that contains a secondary IMU. The ultrasound module functions by aligning the IMU with underlying osseous landmarks, thereby stabilizing sensor placement and minimizing the influence of soft tissue motion. Both IMU modules communicate synchronously, and calibration is achieved through controlled static poses. The combination of ultrasound and IMU data enables more accurate tracking of joint angles, particularly in dynamic scenarios where STA is most pronounced. This system has been purpose-built for clinical scenarios, including ACL injury assessment and rehabilitation monitoring, where portability and precision are critical.

### 2.2. Reference Measurement System

**Figure 1:**
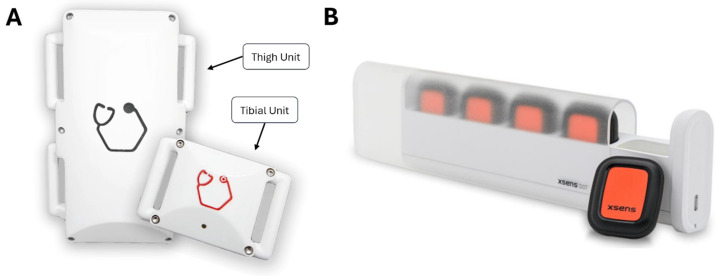
(**A**) GATOR system, comprising the thigh unit (with ultrasound module) and tibial unit. (**B**) Xsens DOTS, serving as the reference measurement system.

Kinematic measurements of the cadaveric knee specimens were obtained using the GATOR system and a reference IMU system (Xsens DOTS). Xsens DOTS was selected as the reference standard in this cadaveric study due to its demonstrated reliability and accuracy in multiple biomechanical investigations [40,41,42,43,44]. In particular, it has shown low root mean square errors (RMSE) below 5° for walking [42,45], along with excellent coefficients of multiple correlation (>0.9886) during repeatability analyses [42], when benchmarked against Vicon systems and models. Owing to its robust performance in gait analysis, sports performance assessments, and clinical evaluations, Xsens DOTS is widely recognized as a dependable wearable IMU platform for lower-limb motion capture [40,41,42,43,44]. By benchmarking GATOR against such an extensively validated system, this study provides a reliable comparison when assessing knee flexion and rotational stability measurements, see Figure 1.

### 2.3. Cadaveric Specimens

A total of 6 unpaired fresh-frozen human cadaveric knees were used in this study. The specimens were confirmed to be free of any prior injuries, surgical history, or gross anatomical abnormalities to minimize confounding factors. Each specimen was stored at −20 °C and thawed at room temperature for 24 h before preparation. To ensure consistency across trials, the femoral and tibial diaphysis were cut 20 cm from the joint line, maintaining anatomical integrity while allowing for secure fixation in the experimental setup. All soft tissues within 10 cm of the joint line were preserved, whereas the remaining soft tissues were removed to expose the tibia, fibula, and femur.

### 2.4. Experimental Setup

#### 2.4.1. Specimen Fixation and Positioning

Steinmann pins (4.5 mm diameter) were inserted into the femur and tibia of six cadaveric specimens using a manual orthopedic drill. To minimize soft tissue forces on the pins, stab incisions were made through the skin and underlying tissues before pin placement. In the tibia, one pin was positioned anterior to the tibial tubercle and another posterior to it, approximately perpendicular to the tibial long axis. Similarly, in the femur, one pin was inserted anterior to the lateral femoral epicondyle and another posterior to it.

Each cadaveric specimen was mounted onto an Oxford Knee Jig, an adjustable clamping system that stabilizes the femur and tibia independently but allows the knee to move with six degrees of freedom [46]. Consequently, flexion and extension are dictated by the native articular surfaces and soft tissues rather than external restraints. During testing, a tensile force was applied to the quadriceps tendon to simulate muscle contraction, and a compressive load was applied proximally to replicate weight-bearing in the cadaveric limb. This fixation method allowed for controlled adjustments of knee flexion and tibial rotation, ensuring reproducible measurements across different experimental conditions.

#### 2.4.2. Instrumentation and Data Acquisition

**Figure 2:**
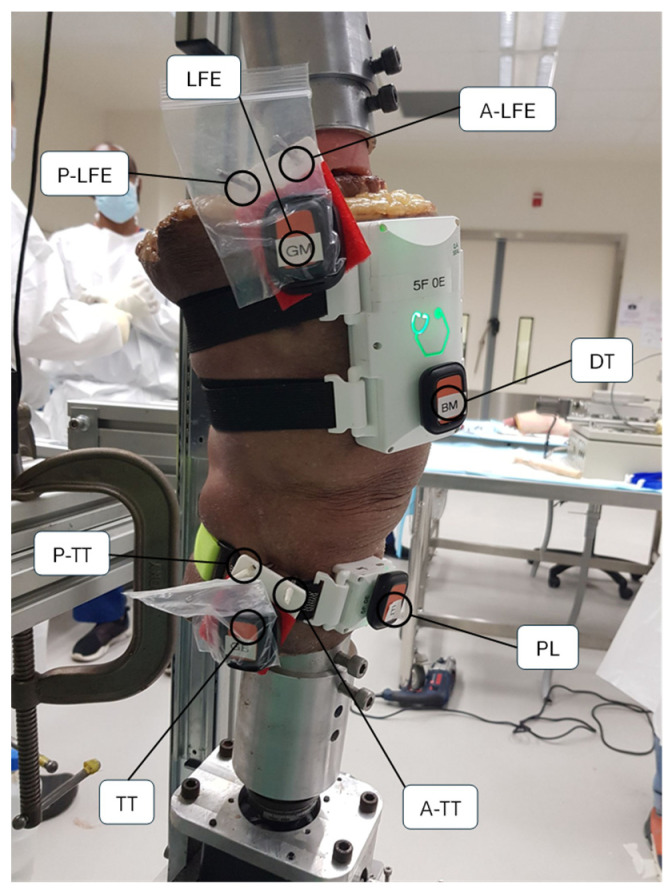
Positions of the Steinmann pins used to mount the Xsens DOTS reference IMUs are shown. Four pins are placed anterior to the tibial tubercle (A-TT), posterior to the tibial tubercle (P-TT), anterior to the lateral femoral epicondyle (A-LFE), and posterior to the lateral femoral epicondyle (P-LFE). Two custom 3D-printed frames connect the respective pairs of tibial (TT) and femoral (LFE) pins, providing stable anchor points for the Xsens DOTS. The GATOR system is secured at the distal thigh (DT) and proximal leg (PL).

Kinematic measurements of the cadaveric knee specimens were captured using two systems: the GATOR system and a reference IMU system (Xsens DOTS). For the GATOR setup, a larger thigh unit was secured to the anterolateral aspect of the distal thigh with elastic straps, while a smaller shank unit was attached to the anterolateral aspect of the proximal leg. The Xsens DOTS reference IMUs, by contrast, were mounted directly onto the intracortical pins using a custom 3D-printed plastic frame that spanned between the pairs of pins on the femur and tibia. This pin-based attachment reduced soft tissue artifact by anchoring the sensors closer to the underlying bone, see Figure 2.

### 2.5. Calibration Procedures

Static calibration poses were recorded to align the measured knee kinematics with the anatomical reference frame. Both the GATOR system and the reference IMU system (Xsens DOTS) were calibrated concurrently in each pose to maintain consistency in angle measurements and data synchronization. In the first pose, the cadaveric knee was positioned in full extension within the Oxford Knee Jig, with both the femur and tibia firmly secured. For the second pose, the knee was placed in slight flexion, and the jig’s base plate was then readjusted to return the knee to full extension. Capturing these two distinct positions helped establish the necessary reference points for accurate sensor alignment and data interpretation.

### 2.6. Experimental Protocols

#### 2.6.1. Experiment A: Measurement of Flexion Range of Motion

Following calibration, each cadaveric knee was initially positioned in full extension within the Oxford Knee Jig, and data recording was initiated while the knee remained stationary. The knee was then gradually flexed in a slow, controlled manner to a predefined angle, where it was held in a stable position. After a brief pause in this flexed position, the knee was carefully extended back to full extension at a controlled pace. Once the knee returned to full extension, it remained stationary before ending the recording. This process was repeated for five trials (n = 5), ensuring consistent and reproducible movements across all experimental runs.

#### 2.6.2. Experiment B: Measurement of Relative Tibial and Femoral Rotation

Following calibration, the cadaveric knee was positioned at each of the predefined flexion angles of 0°, 30°, 60°, 90°, and 120° using the Oxford Knee Jig. These angles were selected to capture a wide range of motion commonly encountered in daily activities and clinical evaluations, extending from near-complete extension (0°) to deep flexion (120°). With the knee held stationary at each chosen flexion angle, data recording was initiated.

**Figure 3:**
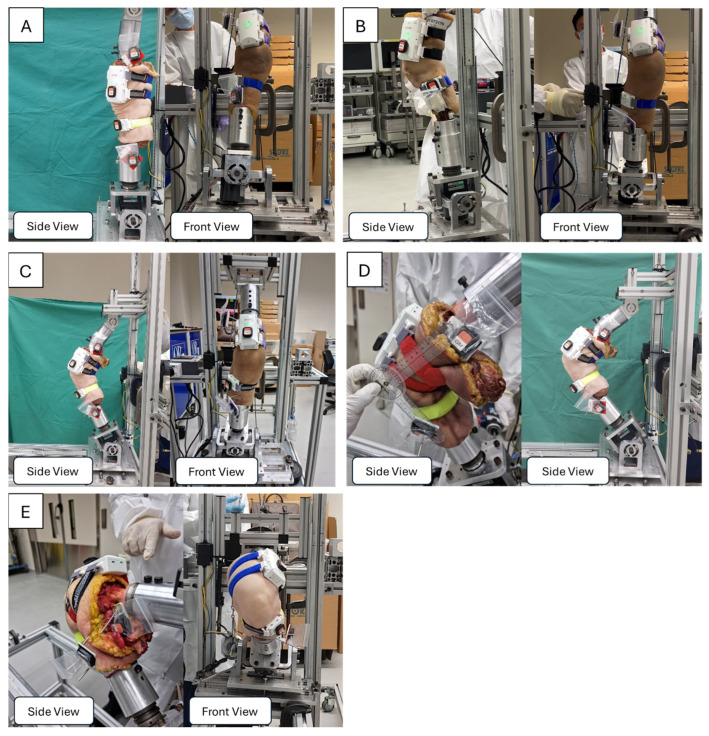
Experimental calibration setup depicting the use of Xsens DOTS and GATOR systems. The cadaveric knee is stabilized in an Oxford Knee Jig, allowing controlled movement in multiple degrees of freedom. The reference Xsens DOTS IMUs are anchored to intracortical pins in alignment with the underlying bone. The tibia is rotated both externally and internally at knee flexion angles of 0° (**A**), 30° (**B**), 60° (**C**), 90° (**D**), and 120° (**E**).

A motorized mechanism then applied an external rotational force to the tibia while the femur remained fixed in place at the selected flexion angle. After the tibia reached its limit of external rotation, it was held briefly before being returned to the starting position. The same procedure was then repeated for internal rotation by reversing the direction of the applied force. Each internal rotation measurement was performed three times, and each external rotation measurement was also repeated three times to ensure reproducibility and account for potential variations in the applied force, see Figure 3.

#### 2.6.3. Statistical Analysis

Data were collected from six cadavers, each undergoing eleven distinct movement protocols. To assess whether measurements followed a normal distribution, the Shapiro–Wilk test was performed. Group means and standard deviations are reported for all movements. Absolute differences between the benchmarked standard Xsens DOTS system and GATOR 3D system were calculated to evaluate measurement accuracy.

Bland–Altman analysis was used to determine the limits of agreement (LoA) between the two measurement methods, with the acceptable LoA defined a priori as being within 5° of no difference [47,48]. In accordance with the 95% standard, most data points were expected to lie within two standard deviations of the mean difference. The results of this analysis are conventionally displayed graphically using a scatter plot by plotting the difference between paired measurements on the Y-axis against the average of those measurements on the X-axis [49,50].

Regression analysis was used to evaluate the relationship between the kinematic data recorded by both systems, where the slope of the regression line and the coefficient of determination (R^2^) were calculated to quantify this association. Sample-to-sample root mean square error (RMSE) and paired *t*-test were performed to evaluate the mean measurements between the Xsens DOTS system and GATOR 3D system. A *p* value of <0.05 is taken to be statistically significant. Bonferroni correction, which adjusts the significance threshold for multiple comparisons, was applied across five knee flexion angles, with a Bonferroni-corrected *p* value of < 0.01 taken to be statistically significant. Random error was quantified to distinguish measurement variability from systematic bias, thereby providing insight into how much of the observed differences could be attributed to random fluctuations. All statistical analyses were conducted using Python 3.9.9.

(1) $$\mathrm{Random}\;\mathrm{Error}={\mathrm{RMSE}}^{2}-{\left(\mathrm{Mean}\;\mathrm{Bias}\right)}^{2}$$

The accuracy and repeatability of the GATOR system were assessed using targeted parameters derived from the analysis. Accuracy was evaluated through the root mean square error (RMSE), which combines systematic and random errors to gauge overall agreement with the benchmark Xsens DOTS system. Lower random error values would indicate minimal measurement variability. For repeatability, the study focused on consistency across trials, reported as the percentage of trials within 5° and 10° of the mean error, % (n), indicating how often GATOR closely aligns with the Xsens measurements.

## 3. Results

Six cadaveric knee specimens successfully completed the full set of motion protocols, which were external rotation, and internal rotation. However, due to an error in Xsens DOTS data capture for one specimen, only data from five cadaveric specimens were included in the final analysis. The Shapiro–Wilk test revealed that the distribution of data was not significantly different from the normal distribution (*p* > 0.05) and normality of our study population was assumed.

**Figure 4:**
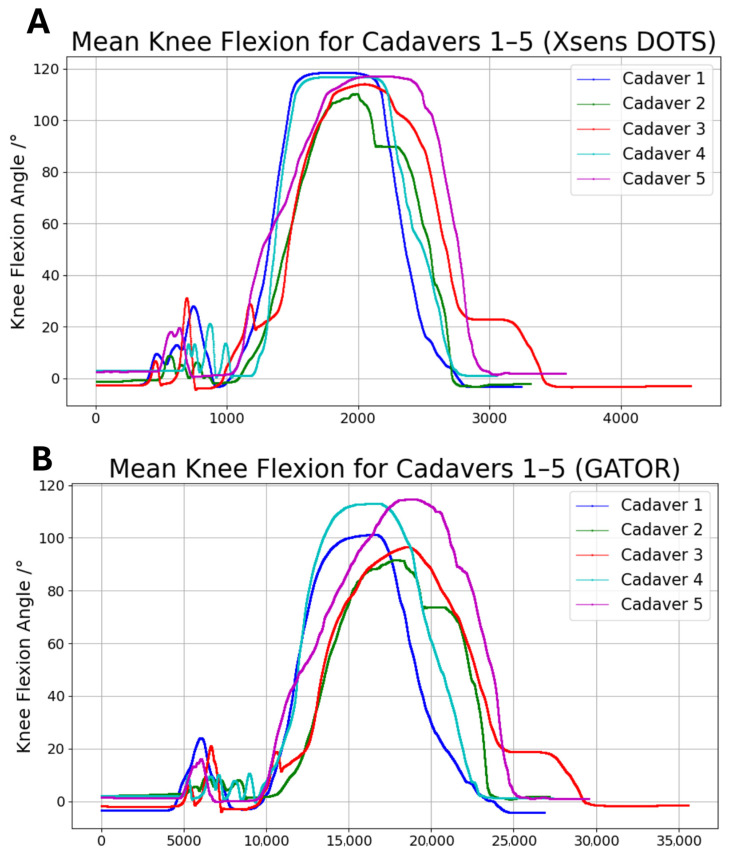
Comparison of mean knee flexion angles measured by (**A**) Xsens DOTS and (**B**) GATOR for cadavers 1 (blue), 2 (green), 3 (red), 4 (cyan), and 5 (purple).

In Experiment A, GATOR measured peak mean knee flexion angles ranging from 91.49° to 114.65°, while Xsens DOTS measured angles ranging from 110.31° to 118.49° (Figure 4).

In Experiment B, the mean bias between Xsens DOTS and GATOR system was smallest at internal rotation (120°) at 1.91°. In contrast, larger values were observed for specific rotation angles, including external rotation (30°) at 6.10° and external rotation (60°) at 6.88° (Table 1). Positive mean biases indicate that the GATOR system appears to underestimate the Xsens DOTS for external and internal rotation, particularly during external rotation at moderate knee flexion angles (30°, 60°, and 90°).

**Figure 5:**
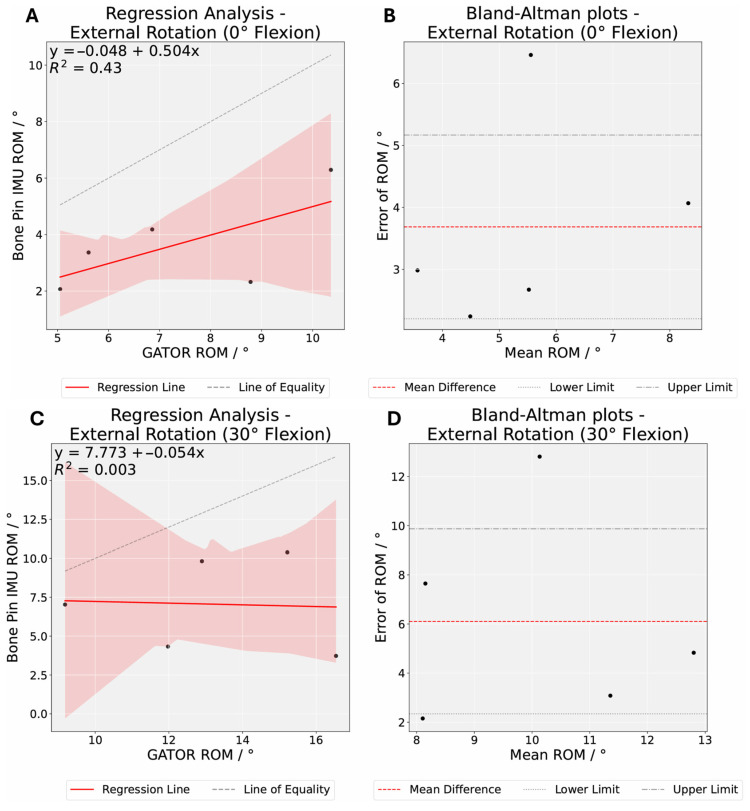

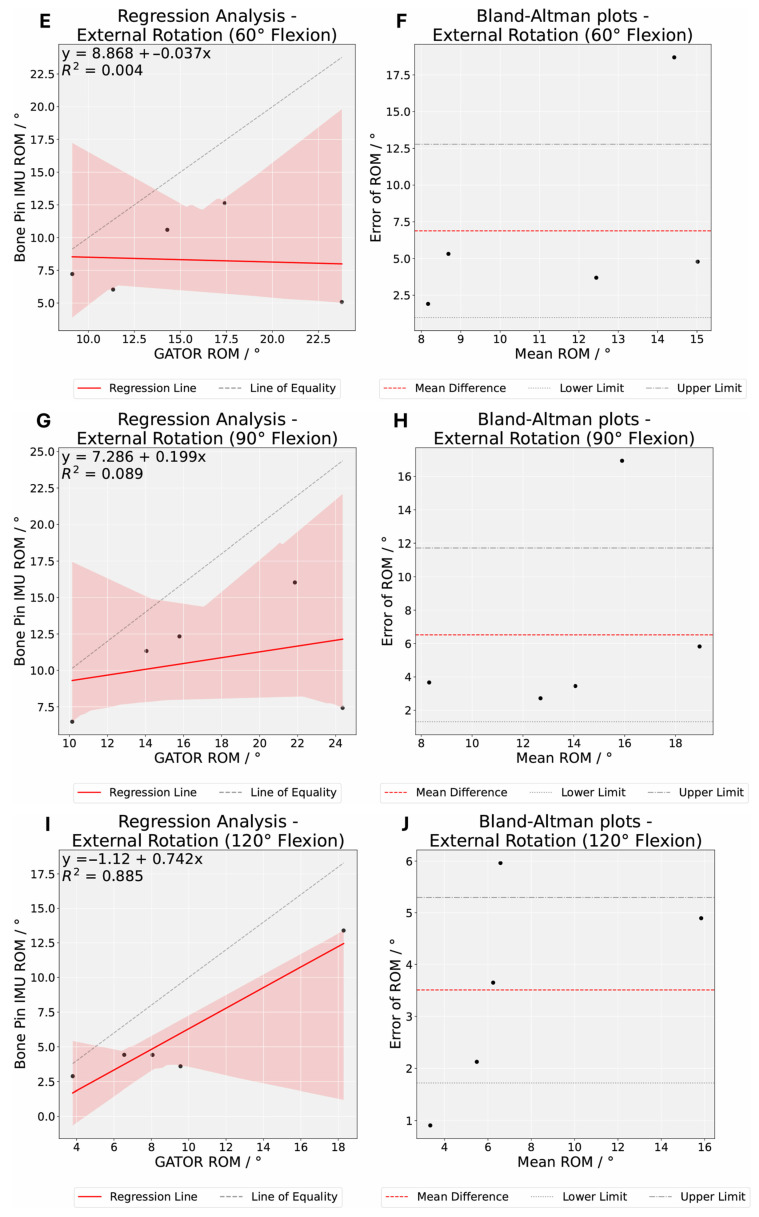

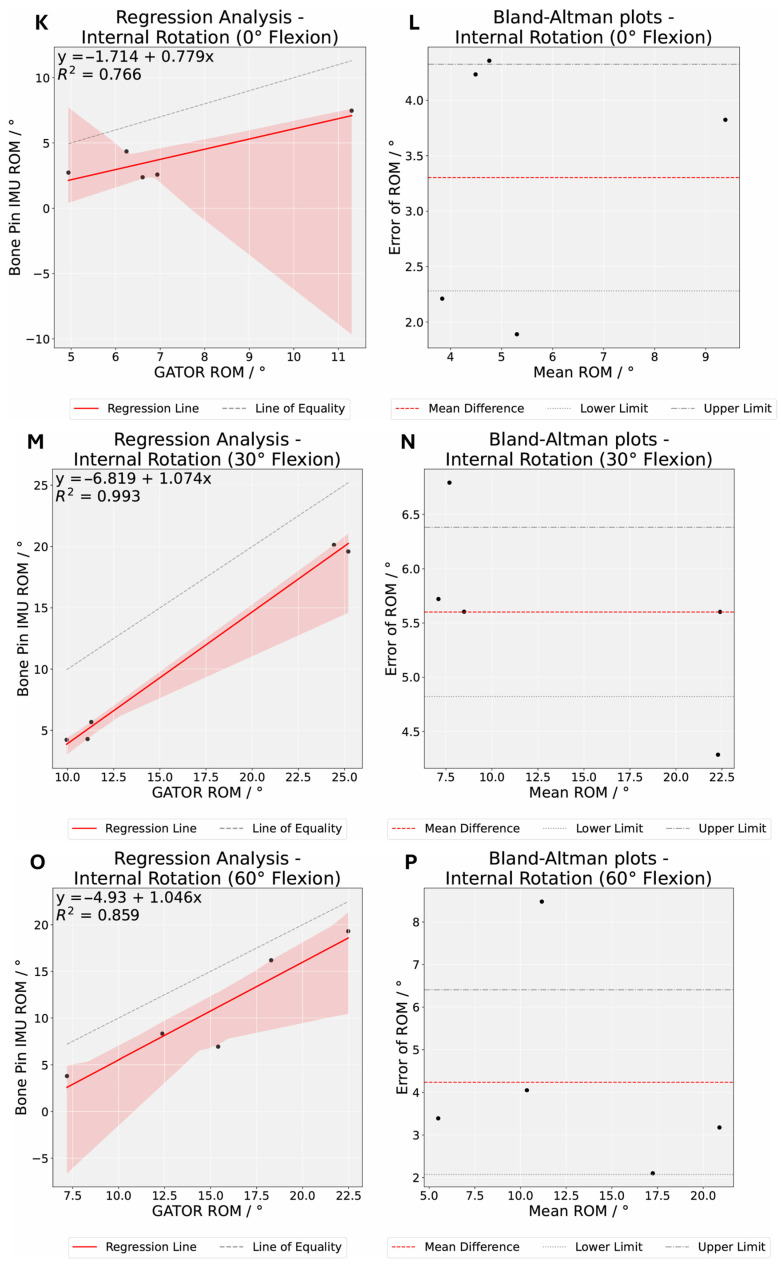

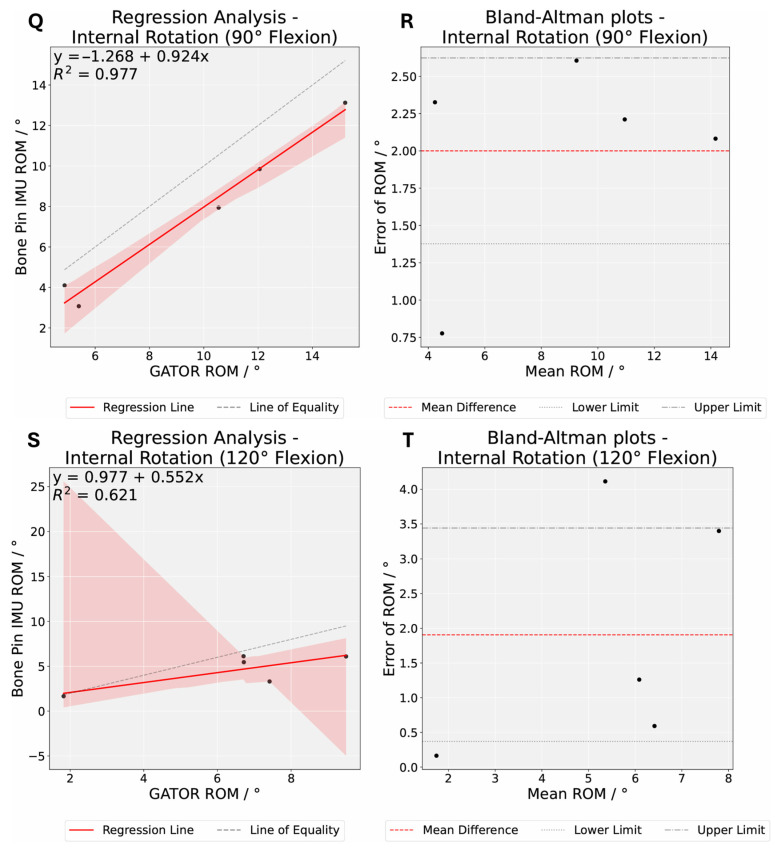
Regression and Bland–Altman analyses comparing knee rotation angle measurements between Xsens DOTS and GATOR systems. Regression analysis describing the correlation of measurements for knee rotation angles between the two systems for external rotation at knee flexion 0° (**A**), 30° (**C**), 60° (**E**), 90° (**G**), and 120° (**I**), and internal rotation at knee flexion 0° (**K**), 30° (**M**), 60° (**O**), 90° (**Q**), and 120° (**S**). Bland–Altman plots demonstrating the difference in knee rotation angles between the two systems for external rotation at knee flexion 0° (**B**), 30° (**D**), 60° (**F**), 90° (**H**), and 120° (**J**), and internal rotation at knee flexion 0° (**L**), 30° (**N**), 60° (**P**), 90° (**R**), and 120° (**T**). The dashed gray lines represent the 95% limits of agreement, corresponding to two standard deviations of the mean difference.

Our Bland–Altman analysis revealed the LoAs greater than 5° of the mean difference on either side for most movements (Figure 5). At least 60% of specimens exhibited a difference of less than 5° between both methods for external and internal rotation, while at least 80% showed a difference of less than 10° (Table 2). Moreover, the initial *t*-tests suggested there were significant differences (*p* < 0.05) for certain conditions (Table 2). However, after a stricter threshold was applied through Bonferroni correction, no statistically significant differences were found for external and internal rotation (P Bonferroni > 0.01), with the exception of internal rotation at knee flexion of 30° (P Bonferroni = 0.002). Higher RMSE and random error values were recorded at external rotation (30°, 60°, and 90°), reflecting greater variability at moderate knee flexion angles (Table 2).

## 4. Discussion

### 4.1. Accuracy and Repeatability of GATOR

The aim of this study was to evaluate the accuracy and repeatability of the GATOR system in measuring knee flexion and rotation relative to a well validated benchmark, Xsens DOTS. In Experiment A, GATOR’s peak mean knee flexion angles (91.49–114.65°) were generally lower than Xsens DOTS’s (110.31–118.49°). In Experiment B, our findings indicate that the GATOR system demonstrates acceptable agreement with the Xsens DOTS system for knee rotation across various knee flexion angles. However, notable discrepancies were observed at moderate flexion angles (30°, 60°, and 90°), particularly during external rotation. Notably, they have higher mean bias (6.1°, 6.52°, 6.88°), higher RMSE (7.21, 8.41, 9.14), and higher random error values (4.29, 5.94, 6.73).

Unlike traditional IMU-based systems that utilize Kalman or Madgwick filters for drift correction [40,41,42,43,44], GATOR leverages ultrasound alignment to maintain sensor positioning relative to bone landmarks, minimizing drift without requiring complex filtering. This bone-referenced placement strategy eliminates the need for computational filtering while providing a fundamentally more stable measurement foundation than IMU-only systems prone to orientation drift. Although a direct comparison between GATOR with and without ultrasound was not performed in this study, the observed improvements in angle accuracy following optimized placement suggest a contributory effect of the ultrasound component. Future studies should isolate and empirically validate this contribution using modular configurations.

**Figure 6:**
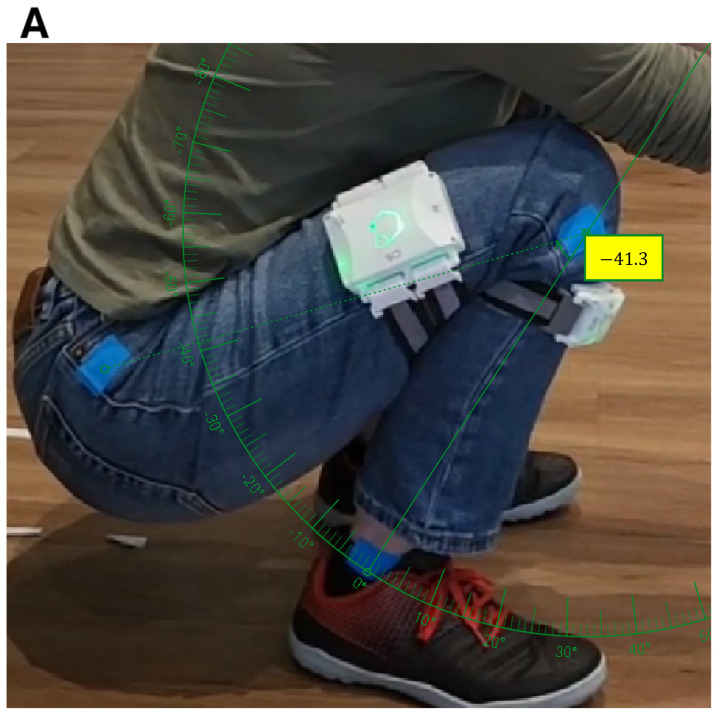

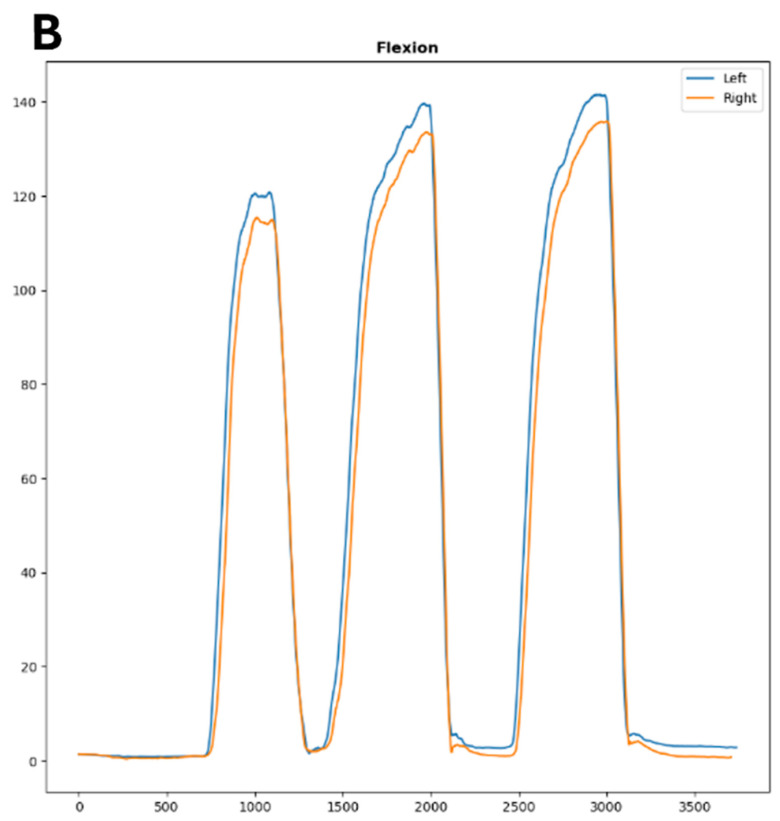
(**A**) Human subject squatting with a measured tibia–femur angle of 41.3°, corresponding to a knee flexion angle of 138.7°. (**B**) Knee flexion time series captured by GATOR for the left (blue) and right (orange) leg.

The inaccuracies in knee flexion may stem from the position of the GATOR devices being too close to the knee joint. This constraint was imposed by the cadavers’ short (20 cm) limb length, which limited the area of placement for the GATOR devices. When the GATOR devices placed near the joint center in cadaveric limbs with limited segment length, the resulting diminished tangential accelerations can lead to an underestimation of total knee flexion and rotation [51,52,53], since a smaller radius yields lower linear acceleration for a given angular motion [54,55]. Several methodological studies emphasize accounting for the IMU’s distance from the joint center, using the sensor’s offset to capture centripetal and tangential accelerations to improve the calculation of internal/external rotation [56]. They recommend strapping the IMUs 10–15 cm away from the knee to maximize sensitivity to rotational motion while minimizing skin movement noise [57]. Using this new insight, we conducted a follow-up test on a human subject with GATOR devices placed approximately 10–15 cm from the knee center, as recommended. When the subject’s knee was flexed to 138.7°, the GATOR measurements precisely matched the known flexion angle, indicating that appropriate sensor placement effectively mitigated the underestimation of knee flexion angles (Figure 6).

As noted in Experiment A, near-pivot sensor placement contributed to the inaccuracies in knee rotation angle measurements observed in Experiment B [51,52,58]. Furthermore, Steinmann pins inserted near the tibial tubercle and lateral femoral epicondyle may introduce subtle mechanical constraints that disproportionately affect external rotation at moderate knee flexion angles. Although these pins are meant to provide stable, bone-anchored reference points, their rigidity can unintentionally restrict or alter the knee’s natural rotation arc under quadriceps tension and weight-bearing loads [54]. This combination of near-pivot sensor placement and possible pin-induced mechanical resistance helps explain why external rotation measurements exhibit greater mean bias and random errors compared to internal rotation, with pronounced deviations at moderate knee flexion angles.

Nevertheless, our results show that random errors for internal rotation at knee flexion angles (0°, 30°, 90°, 120°) remained below 1.75°, while external rotation at moderate flexion angles exhibited considerably larger variability and an extended error range [59]. This observation, in conjunction with the prior discussion of near-pivot sensor placement and Steinmann pins, suggests that mechanical constraints near the femoral pins, rather than soft-tissue movement, were primarily responsible for these discrepancies. GATOR’s ultrasound-based alignment effectively stabilizes the distal sensor under varying joint configurations, indicating minimal soft-tissue interference around the tibia.

Additionally, our findings show a consistent positive mean bias for both external and internal rotation (Xsens DOTS IMU Mean–GATOR Mean), presumably due to the systematic overestimation of Xsens DOTS. This outcome aligns with the phenomenon of IMU drift, where integration of small errors in gyroscope and accelerometer measurements accumulate over time, causing incremental deviations from the true orientation or motion [40,54,55,56,60]. As a result, the IMU’s internal orientation estimate can drift in a single direction due to uncompensated gyro bias [61]. Although Xsens DOTS employs an onboard Kalman filter (XKF) to mitigate drift [40], any residual bias or imperfect magnetometer correction can gradually inflate measured rotation angles. These findings concur with Bailey et al. [62], who reported an overestimation of 3.1–6.8° in lower-limb rotations using Xsens DOTS, closely matching the magnitudes we observed. Consequently, the accumulated drift manifests as a consistently higher measurement in Xsens DOTS, resulting in a positive bias.

Overall, the accuracy and repeatability of the GATOR system appears consistent with current research on IMU-based knee rotation measurements. Few studies have specifically investigated knee internal/external rotation using IMU-based systems, as most literature primarily focuses on flexion-extension or adduction-abduction. Moreover, no existing work has employed an IMU-and-ultrasound fusion approach for measuring knee rotation, highlighting the uniqueness of our study. Nevertheless, existing IMU-versus-OMC studies provides a comparative framework: Xavier et al. reported a mean bias of 4.7° and an RMSE of 5.2–5.6° in external-internal rotation during low walking velocities [63], while Nichols et al. observed a mean bias of 3.9° and an RMSE of 4.6° in jump landing tasks [64]. Similarly, Weber et al. noted an RMSE of 6.5° under walking conditions [65], while Sagasser et al. observed 8.6° for external–internal rotation in a cadaveric setup [66]. In our study, GATOR exhibited an RMSE of 6.9° for external rotation, 3.93° for internal rotation, and 5.61° for external–internal rotation; mean biases ranged from 1.91° to 5.6° for internal rotation and 3.51° to 6.88° for external rotation (Table 3). These results align with the mean bias (3.9–4.7°) and RMSE (4.6–8.6°) ranges documented in previous IMU-versus-OMC knee rotation studies [64,65,66]. This suggests that knee joint rotation angles can be assessed in a repeatable, reliable, and reproducible manner using the GATOR system.

### 4.2. Limitations

Traditionally, most IMU-based knee kinematics studies employ three or four sensors, typically mounted on the thigh, shank, and waist [23,60,67,68], to capture multi segment motion. Although Favre et al. introduced a two IMU-configuration fixed on the thigh and shank in 2008 [69], relatively few modern approaches have adopted similarly minimal IMU setups [64,65]. While we recognize that additional IMUs can improve angular measurement accuracy and reduce redundancy [60,63,69,70,71,72], their added complexity diminishes portability and movement freedom. Being sufficiently compact to mount on the body without impeding motion, while remaining simple enough for deployment beyond laboratory settings [69], is crucial for the usability and compliance of GATOR in clinical environments and remote rehabilitation. However, the physiological range of tibial internal/external rotation at the knee is limited. For instance, Burford Jr et al. reported internal/external rotation angles of 5.03–17.09° in normal cadaveric knees [72]. Classic biomechanical studies also show that external and internal rotation rarely exceeds 10–15° [44,69,73,74]. Given the knee’s geometric constraints and ligamentous support, the small magnitude of rotation makes the addition of IMUs less essential. Moreover, Steinmann pins inserted near the tibial tubercle and lateral femoral epicondyle may introduce subtle mechanical constraints that affect measurement accuracy, particularly for external rotation at moderate knee flexion angles. Additionally, near-pivot sensor placement and potential variability in soft tissue artifacts across different limb lengths and body compositions represent further limitations. Future studies should isolate the ultrasound component’s contribution to STA reduction using modular configurations and quantify STA across diverse populations. Overall, GATOR’s two IMU configurations, integrated ultrasound imaging, strikes a favorable balance between measurement accuracy, user compliance, and practical deployment outside the laboratory setting.

### 4.3. Potential Clinical Implications

GATOR’s validated accuracy in assessing tibial rotation carries notable implications for the diagnosis, management, and rehabilitation of knee injuries. By providing precise rotational data with minimal soft tissue artifact, the device offers clinicians a more reliable means of identifying subtle rotational instabilities common in ligamentous pathologies such as anterior cruciate ligament (ACL) tears. In pre-operative assessments, detailed rotational profiles can guide surgical planning, while post-operative monitoring can help determine whether grafts or reconstructive procedures have restored appropriate joint mechanics. This objective feedback allows clinicians to refine rehabilitation timelines and adjust exercise regimens to optimize healing, thus facilitating more effective and individualized care.

Beyond acute injury management, GATOR’s capabilities also hold value in the long-term evaluation of post-surgical outcomes and athletic performance. The system’s consistent measurements enable regular screening for residual or developing biomechanical deficiencies, ensuring that rehabilitative interventions remain targeted and evidence based. Athletes recovering from knee injuries stand to benefit from ongoing rotational assessments, which can reveal early indications of compromised function or compensatory movement patterns that might predispose them to re-injury. In this way, GATOR’s robust kinematic data offer a pathway toward more precise return-to-play decisions and a safer, progressive transition from clinical recovery to full athletic participation.

## 5. Conclusions

Based on our controlled laboratory findings, we conclude that the GATOR system is accurate and reliable for measuring knee flexion and rotation. Its minimal soft tissue artifact and ultrasound fusion approach enable fine rotational detail, especially with sensors placed 10–15 cm from the knee center. These results highlight GATOR’s potential as a practical, portable solution for assessing knee rotational stability and facilitating targeted interventions in both clinical and rehabilitation settings.

Future investigations should systematically evaluate the extent of pin-induced mechanical restrictions across a range of knee flexion and rotation angles. In parallel, further studies should quantify soft tissue artifacts (STA) associated with GATOR, particularly across varying limb lengths and body compositions.

## Figures and Tables

**Table 1:** Bland–Altman analysis: Xsen Dots system vs. GATOR system.

Exercise, °	Xsen Dots IMU Mean, ° (SD ^3^)	GATOR Mean, ° (SD)	Mean Bias ^1^, ° (SD)	LoA ^2,4^ 95% CI ^5^, °
External Rotation (0)	7.33 (1.98)	3.65 (1.52)	3.69 (1.69)	[2.20, 5.17]
External Rotation (30)	13.16 (2.57)	7.06 (2.73)	6.10 (4.29)	[2.34, 9.87]
External Rotation (60)	15.19 (5.12)	8.31 (2.85)	6.88 (6.73)	[0.98, 12.78]
External Rotation (90)	17.24 (5.19)	10.72 (3.46)	6.52 (5.94)	[1.31, 11.72]
External Rotation (120)	9.25 (4.90)	5.74 (3.87)	3.51 (2.04)	[1.72, 5.30]
Internal Rotation (0)	7.20 (2.16)	3.90 (1.92)	3.30 (1.17)	[2.28, 4.33]
Internal Rotation (30)	16.39 (6.90)	10.79 (7.44)	5.60 (0.89)	[4.82, 6.38]
Internal Rotation (60)	15.15 (5.19)	10.91 (5.86)	4.24 (2.47)	[2.07, 6.41]
Internal Rotation (90)	9.62 (3.96)	7.62 (3.70)	2.00 (0.71)	[1.38, 2.62]
Internal Rotation (120)	6.43 (2.52)	4.53 (1.77)	1.91 (1.75)	[0.37, 3.44]

^1^ Mean bias = Xsens Dots—GATOR measurement. ^2^ LoA = mean difference ± 1.96 (SD difference). ^3^ SD, standard deviation. ^4^ LoA, limit of agreement. ^5^ CI, confidence interval.

**Table 2:** Additional validation analysis: Xsen Dots system vs. GATOR system.

Exercise, °	Percentage of Trials Within 5° of Mean Error, % (n ^1^)	Percentage of Trials Within 10° of Mean Error, % (n ^1^)	R^2^	RMSE ^2^, °	P	P Bonferroni	Random Error
External Rotation (0)	80.0 (4)	100.0 (5)	0.43	3.98	0.008	0.09	1.69
External Rotation (30)	60.0 (3)	80.0 (4)	0.0026	7.21	0.034	0.369	4.29
External Rotation (60)	60.0 (3)	80.0 (4)	0.0044	9.14	0.084	0.927	6.73
External Rotation (90)	60.0 (3)	80.0 (4)	0.09	8.41	0.07	0.771	5.94
External Rotation (120)	80.0 (4)	100.0 (5)	0.88	3.95	0.018	0.202	2.04
Internal Rotation (0)	100.0 (5)	100.0 (5)	0.77	3.46	0.003	0.035	1.17
Internal Rotation (30)	20.0 (1)	100.0 (5)	0.99	5.66	0.0	0.002	0.89
Internal Rotation (60)	80.0 (4)	100.0 (5)	0.86	4.78	0.019	0.204	2.47
Internal Rotation (90)	100.0 (5)	100.0 (5)	0.98	2.1	0.003	0.036	0.71
Internal Rotation (120)	100.0 (5)	100.0 (5)	0.62	2.47	0.072	0.791	1.75

^1^ n, number of cadavers. ^2^ RMSE, root mean square error.

**Table 3:** RMSE for external and internal rotation of the knee.

Exercise Type	RMSE
External Rotation	6.90
Internal Rotation	3.93
External-Internal Rotation	5.61

## Data Availability

The original contributions presented in this study are included in the article. Further inquiries can be directed to the corresponding author(s).

## Abbreviations

The following abbreviations are used in this manuscript:

ACL	Anterior Cruciate Ligament
IMU	Inertial Measurement Unit
STA	Soft Tissue Artifact
RMSE	Root Mean Square Error
LoA	Limits of Agreement
R^2^	Coefficient of Determination
SD	Standard Deviation
CI	Confidence Interval
